# A conceptual framework of computations in mid-level vision

**DOI:** 10.3389/fncom.2014.00158

**Published:** 2014-12-12

**Authors:** Jonas Kubilius, Johan Wagemans, Hans P. Op de Beeck

**Affiliations:** ^1^Laboratory of Biological Psychology, Faculty of Psychology and Educational Sciences, KU LeuvenLeuven, Belgium; ^2^Laboratory of Experimental Psychology, Faculty of Psychology and Educational Sciences, KU LeuvenLeuven, Belgium

**Keywords:** mid-level vision, similarity, pooling, perceptual organization, summary statistics

## Abstract

If a picture is worth a thousand words, as an English idiom goes, what should those words—or, rather, descriptors—capture? What format of image representation would be sufficiently rich if we were to reconstruct the essence of images from their descriptors? In this paper, we set out to develop a conceptual framework that would be: (i) biologically plausible in order to provide a better mechanistic understanding of our visual system; (ii) sufficiently robust to apply in practice on realistic images; and (iii) able to tap into underlying structure of our visual world. We bring forward three key ideas. First, we argue that surface-based representations are constructed based on feature inference from the input in the intermediate processing layers of the visual system. Such representations are computed in a largely pre-semantic (prior to categorization) and pre-attentive manner using multiple cues (orientation, color, polarity, variation in orientation, and so on), and explicitly retain configural relations between features. The constructed surfaces may be partially overlapping to compensate for occlusions and are ordered in depth (figure-ground organization). Second, we propose that such intermediate representations could be formed by a hierarchical computation of similarity between features in local image patches and pooling of highly-similar units, and reestimated via recurrent loops according to the task demands. Finally, we suggest to use datasets composed of realistically rendered artificial objects and surfaces in order to better understand a model's behavior and its limitations.

## Vision as an image understanding system

The visual system of primates processes visual inputs incredibly rapidly. Within 100 ms observers are capable of reliably reporting and remembering contents of natural scenes (e.g., Potter, 1976; Thorpe et al., 1996; Li et al., 2002; Quiroga et al., 2008). Such fast processing puts tight constraints on models of vision as most computations should be done roughly within the first feed-forward wave of information. Efforts to understand how this is possible have led to the so-called standard view of the primate visual system where objects are rapidly extracted from images by a hierarchy of linear and non-linear processing stages, where simple and specific features are combined in a non-linear fashion, resulting in increasingly more complex and more transformation-tolerant features (Fukushima, 1980; Marr, 1982; Ullman and Basri, 1991; Riesenhuber and Poggio, 1999; DiCarlo and Cox, 2007; DiCarlo et al., 2012; see Kreiman, 2013, for a review).

In particular, in primate visual cortex the earliest stages of visual processing are thought to act as simple local feature detectors. For example, retinal ganglion and lateral geniculate nucleus cells preferentially respond to blobs with center-surround organization (Kuffler, 1953; Hubel and Wiesel, 1961), while neurons in primary visual area V1 respond to oriented edges and bars (Hubel and Wiesel, 1962; see Carandini et al., 2005, for a review). These detectors act locally (within their receptive field) and thus are very sensitive to changes in position or size. In contrast, neurons in the final stages of visual processing in the inferior temporal cortex respond to complex stimuli, including whole objects (Tanaka, 1996; Kourtzi and Kanwisher, 2001; Op de Beeck et al., 2001; Huth et al., 2012), faces (Desimone et al., 1984; Kanwisher et al., 1997; Tsao et al., 2006), scenes (Epstein and Kanwisher, 1998; Kornblith et al., 2013), bodies (Downing et al., 2001; Peelen and Downing, 2005) and other categories. At this stage, neurons have large receptive fields and thus are tolerant to changes in position, size, orientation, lighting, and clutter (DiCarlo and Cox, 2007). While the exact details of the properties of neurons at the low and high visual areas remain an area of active research, in our view the most puzzling question is the following: What computations are performed at the intermediate steps of information processing in order to bridge simple local early representations to highly multidimensional representations of objects and scenes?

In primates, inspired by Hubel and Wiesel's (1965) proposal of the hierarchical processing in the visual cortex, a number of studies focused on demonstrating sensitivity to the increasing complexity of features along the visual hierarchy. For example, in V2 angle or curvature detectors have been reported (Dobbins et al., 1987; Ito and Komatsu, 2004). In V4, neurons are sensitive to even more complex curved fragments and three-dimensional parts of surfaces (Pasupathy and Connor, 1999, 2001, 2002; Yamane et al., 2008). Thus, the idea is that intermediate layers are responsible for gradually combining simpler features into more complex ones (Riesenhuber and Poggio, 1999; Rodríguez-Sánchez and Tsotsos, 2012).

However, building a system that could robustly utilize such a connection scheme on natural images is difficult. On the one hand, combining simpler features into more complex ones is complicated due to the presence of clutter. Robust mechanisms are necessary to combine the “correct” features and leave out the noise. Similarly, in order to detect complex features, enormous dictionaries must be built since the number of possible feature combinations is huge, so this process is highly resource-intensive (but see Fidler et al., 2009, for an inspiring approach to the issue). On the other hand, focusing solely on edges and their combinations into shapes misses a number of other useful cues in the images—such as differences in color, texture, motion and so on—and thus may lack the necessary power both to process object shapes and to be useful for other tasks that the visual system is performing (e.g., interaction with objects in a scene, navigation, or recovering spatial layout; Regan, 2000).

Thus, in computer vision, partially due to the described limitations of the standard view of primate visual system and partially due to the development of robust algorithms for dealing with large numbers of features, the actually implemented models of vision have bypassed thinking about intermediate representations altogether in their implementations. Instead, such models rely solely on the established features of V1 (namely, oriented edge detection) and directly apply sophisticated machine learning techniques (such as support vector machines) to detect what object categories are likely to occur in the given image. Somewhat surprisingly, this idea works very well for a number of complex tasks. For example, in the famous algorithm by Viola and Jones (2001), faces are detected using several simplistic feature detectors, reminiscent of the odd and even filters of V1. In Oliva and Torralba's GIST framework (2001, 2006; Torralba and Oliva, 2003), scene categorization is achieved by computing global histogram statistics of oriented filter outputs. Flat architectures of SIFT (Lowe, 2004) or HoG (Dalal and Triggs, 2005) that largely rely on oriented feature detection have seen a wide adoption for a variety of visual tasks in computer vision, and, in combination with multi-scale processing (Bosch et al., 2007), for a long time these models that have no hierarchies have been the state-of-the-art approach.

However, eventually hierarchical models that contain intermediate representations ultimately proved superior in many complex visual tasks. While such deep networks have been proposed several decades ago, (Fukushima, 1980; LeCun et al., 1989; Schmidhuber, 1992), only recently upon development of more robust procedures for learning from large pools of data (Hinton and Salakhutdinov, 2006; Boureau et al., 2010) such networks managed to achieve state-of-the-art object identification performance on demanding datasets that contain millions of exemplars, such as the Large Scale Visual Recognition Challenge (Deng et al., 2009; Krizhevsky et al., 2012; Sermanet et al., 2013; Szegedy et al., 2014), or that demand fine-grain discrimination as in the case of face recognition (Lu and Tang, 2014; Taigman et al., 2014). Moreover, these networks have been reported to perform extremely well on a number of visual tasks (Razavian et al., 2014). While many challenges remain (Russakovsky et al., 2013), the fact that base-level object categorization and localization have been very successful and in some cases even approaching or superseding human-level performance (Serre et al., 2007; Lu and Tang, 2014; Taigman et al., 2014) is greatly encouraging. Importantly, representations learned by such deep networks have been shown to match well the representations in the primate V4 and IT (Yamins et al., 2014), demonstrating the relevance of these models to understanding biological vision.

Naturally, the success of these object recognition models begs the question whether we now understand how the visual system processes images. It is tempting to conclude that weakly organized collections of features are sufficient for object and scene categorization, and, by extension, scene understanding. However, it is important to realize that, engineering advances aside, each layer in these architectures is based on the same principles characterized in the early visual processing of the primate brain. Is there really nothing more going on in the intermediate stages of processing?

In the following section, we consider what the computational goal of mid-level vision might be (cf. Marr, 1982). Based on these insights, in Section “Intermediate Computations” we propose basic computational mechanisms that we hypothesize to be sufficient to account for processes occurring at intermediate stages. Finally, we discuss what model evaluation procedures could help in guiding the implementation of such a system.

## What do mid-level visual areas do?

### Feature interpolation

Typically, a model of vision is operationalized as a feature extraction system. Features that are present in the input image need to be detected, so that a veridical (or at least useful) representation of the world (or objects in it) can be reconstructed. However, visual inputs are necessarily impoverished (e.g., due to collapsing of the third dimension as the image is projected on the retina), incomplete (e.g., due to some objects partially occluding others), ambiguous (e.g., due to shadows), and noisy. As a consequence, the problem of vision is not only feature detection but also feature inference (Purves et al., 2014).

**Figure 1:**
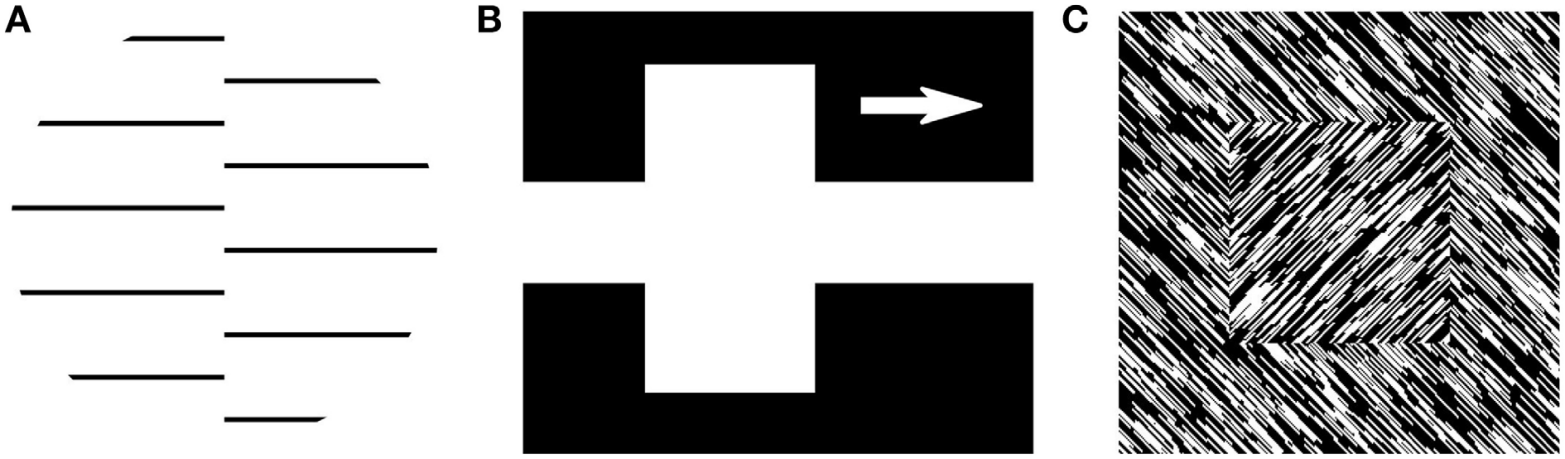
**Feature interpolation. (A)** A second-order boundary stimulus as used by von der Heydt et al. (1984). **(B)** A stimulus with an illusory contour is perceived in the white gap between the two parts of the white rectangle, as used by von der Heydt et al. (1984). The arrow indicates that the white rectangle was moving. **(C)** A stimulus where a shape is defined entirely by second-order cues (that is, a difference in orientation), used in many figure-ground segmentation studies (e.g., Lamme, 1995).

A number of studies have shown that mid-level vision is heavily involved in feature inference. Consider, for example, the seminal series of studies by von der Heydt et al. (1984), von der Heydt and Peterhans (1989), who compared neural responses to the typical luminance-defined stimuli and the neural responses to the same stimuli defined by cues other than luminance. In one of their conditions, a stimulus was composed of two regions containing line segments but with one region shifted with respect to the other, forming an offset-defined discontinuity in the texture, which we refer to as a second-order edge (Figure 1A). Importantly, a simple edge-detecting V1 model would not be able to find such edges, so if some neurons in the visual cortex were responding to such stimuli, it would mean that a higher-order computation is at work that somehow is capable of integrating information across the two regions in the image.

Consistent with the known properties of early visual areas, the researchers observed a robust response to the luminance-defined edges. However, in addition they also demonstrated that some neurons in V2 responded to the second-order edges, and, in fact, often with the same orientation preference as to luminance-defined edges. Moreover, Lamme et al. (1999) reported that V1 neurons were also responding to this boundary roughly 60 ms after stimulus onset and suggested iso-orientation suppression as a mechanism behind such fast second-order edge detections. These findings have since been replicated in V2 and V4 (Ramsden et al., 2001; Song and Baker, 2007; El-Shamayleh and Movshon, 2011; Pan et al., 2012) and also reported for discontinuities in orientation (Larsson et al., 2006; Allen et al., 2009; Schmid et al., 2014), motion (Marcar et al., 2000), and contrast (Mareschal and Baker, 1998; Song and Baker, 2007; Li et al., 2014). Taken together, these findings demonstrate that even in the absence of luminance-defined borders in the inputs, mid-level areas infer potential borders from differences in other cues. Importantly, this operation is different from the typical feature detection and combination scheme because in this case a feature is computed that is not present in the input (that is, a second-order border).

An even more extreme example of such feature inference has been demonstrated by another condition in von der Heydt and colleagues' experiments where they used a stimulus inspired by the Kanizsa triangle (Kanizsa, 1955). The stimulus was defined as a white bar moving over two black bars, separated by a white gap (Figure 1B)—thus, although physically there were no edges connecting the two halves of the white bar, subjectively observers would nonetheless report seeing the complete white bar, effectively interpolating its borders or surface across the white gap. We refer to such borders as illusory contours. Surprisingly, for this condition, von der Heydt et al. (1984) also reported neurons in V2 responding to these illusory contours, and, in fact, nearly as vigorously as to the luminance-defined ones.

**Figure 2:**
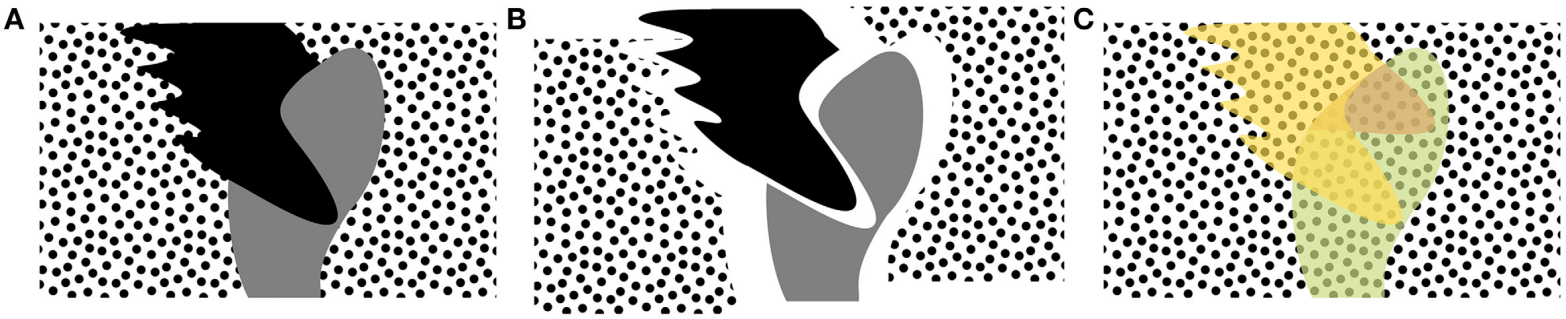
**Seeing is not the same as perceiving**. Observers report perceiving the configuration in **(A)** to be composed of full shapes as depicted in **(C)** rather than as in **(B)** which reflects the physical inputs where shapes are fragmented and two portions of background are separate. In **(C)**, the gray shape has been interpolated behind the black shape (depicted in green), indicating that mapping of a two-dimensional surface in a three-dimensional space is already necessary to represent depth relations. Furthermore, the background is also a single surface rather than two separate regions and also with its statistical properties (polka dot pattern) filled in. Some observers will also see the black shape interpolated behind the gray one (depicted in red), but this percept is much less consistent among observers than the completion of the gray shape behind the black one, indicating that surface inference might not be precise and rather indicate probabilities of possible contour and surface properties.

If these examples appear only as curious cases of feature inference in artificial setups, imagine a typical cluttered image where multiple objects are partially occluded. Just like in the two previous cases, the visual system appears to interpolate occluded parts of objects at the early stages of visual information processing (a process known as amodal completion; van Lier et al., 1994; Ban et al., 2013). For example, Figure 2A is interpreted as a gray blobby shape partially occluded by the black blobby shape, both on a dotted background, as in Figure 2C. In fact, we cannot help but perceive the gray shape inferred behind the black occluder and our phenomenology is most certainly not captured by segmentation into separate non-overlapping regions as in Figure 2B.

Similarly, the background appears to continue behind the two shapes even though there is no physical connection between the left and the right portion of it, demonstrating that filling-in is not confined to objects but applies in a more generic manner to any occluded region in the input. Moreover, at least phenomenologically, this filling-in appears to involve not only surface interpolation but also the spread of feature statistics. In our example, observers would report that the occluded part of the background is likely to continue the pattern of polka dots (van Lier, 1999).

Moreover, just like in the other two cases (second-order borders and illusory contours), the amodal interpolation has been reported to be established relatively fast, already in 75–200 ms (Sekuler and Palmer, 1992; Ringach and Shapley, 1996; Murray et al., 2001; Rauschenberger et al., 2006), and has also been observed in the early modulation of the occluded parts of shapes in monkey V4 (Bushnell et al., 2011; Kosai et al., 2014).

Taken together, we see that the visual system actively performs feature inference and it is an early process that may be initiated already with the first wave of information. It is important to note that in all of these cases, the inference does not necessarily produce a complete feature or a shape. Rather, it may reflect a rough estimate of statistical properties of the shape (cf. “fuzzy completions,” van Lier, 1999) or the probability of possible completions where the missing part of the shape may occur (D'Antona et al., 2013).

### Relational information and surface construction

But what is the purpose of feature extraction or interpolation? In many object recognition models, for example, the extracted features are used directly to perform categorization. Notice that such an output lacks the explicit assignment of the features to one object or another, that is, object shapes are not explicitly represented. Such model behavior is strikingly at odds with our phenomenology dominated by explicit object shapes or surfaces. This idea has been nicely illustrated by Lamme (1995) who investigated neural responses to a shape entirely defined by a second-order boundary. His stimulus consisted of a field of oriented noisy elements embedded in a background of an opposite orientation (Figure 1C). In order to perceive this shape, the visual system must be able to (i) infer second-order borders and (ii) combine them into the shape as a whole. Lamme (1995) showed that neurons in monkey V1 with receptive fields inside that shape reliably respond more than those outside, that is, the visual system explicitly represents where the figure is. Moreover, the observed enhancement was not instantaneous but rather developed in three stages (as described in Lamme et al., 1999). Early on, only responses to local features were observed. Within a 100 ms, responses to the second-order boundary emerged. Finally, neurons in V1 corresponding to the figural region of the display started responding more than the background. This effect was later shown to be the effect of feedback from higher visual areas such as V4, where such figure-ground assignments are thought to emerge (Poort et al., 2012). Taken together, this example demonstrates that the visual system gradually extracts not only the contour of a shape but also its inside, resulting in a full surface reconstruction.

More broadly, it has been argued that surface-based representations form a critical link between early- and high-level computations (Nakayama et al., 1995; see also Pylyshyn, 2001). Moreover, the presence of a surface strongly influences even the earliest computations of the visual information processing such as the iso-orientation suppression (Joo and Murray, 2014). Finally, surface-based representations can also be beneficial for object identification tasks because surfaces are topologically stable structures and thus largely invariant to affine transformations (Chen, 1982, 2005). For example, a hole in a surface remains present despite drastic changes in its position, orientation or rotation in depth, or to the changes in surface structure (Chen, 1982; Todd et al., 2014).

In general, we argue that encoding spatial relations—whether between features, or deciding which features belong to the same object or surface, or ordering the surfaces in space—provides a tremendous wealth of information (Biederman, 1987; Barenholtz and Tarr, 2007; Oliva and Torralba, 2007): Knowing that a car is on the road or above the road makes a big difference, but using only features without relations between them might fail to capture these differences (Choi et al., 2012). One influencial account of the power of spatial relations has been provided by Biederman (1987), who noticed that certain spatial relations between features, known as non-accidental properties, remain largely invariant to affine transformations in space. For example, short parallel lines nearly always remain parallel despite changes in viewpoint. He proposed that these relations might be used to encode different object categories, and later Hummel and Biederman (1992) developed a model illustrating how such a system might work. While the exact purpose of such structural representations in recognition has been heavily debated since (Barenholtz and Tarr, 2007), consistent with this idea a number of studies demonstrated that observers are very sensitive to changes in these invariant features of a shape (Wagemans et al., 1997, 2000; Vogels et al., 2001; Kayaert et al., 2005a,b; Lescroart et al., 2010; Amir et al., 2012).

Similarly, Feldman (1997, 2003) and van Lier et al. (1994) argued that configural regularities of the inputs are used to organize features into objects, and human visual system has been shown to be sensitive to such configural relations (Kubilius et al., 2014). Moreover, Blum (1973) proposed that the configuration of shapes is encoded in the visual system by representing their skeletal, or medial axis, structure, and Hung et al. (2012) showed that neurons in monkey IT indeed respond both to the contour of a shape and its medial axis structure. Taken together, these studies highlight the fact that the visual system utilizes configural relations between features and surfaces in the higher visual areas, and therefore an explicit encoding of these relations should be supported by mid-level computations.

### Representations for multiple tasks, not only object recognition

We argued that mid-level vision was involved in feature detection and surface construction, such that in the end the shape of an object could be reliably extracted from the image. However, the long quest for superior object identification algorithms has somehow overshadowed the fact that visual cortex can achieve more than just object identification. Vision is our means to understanding the world, whereas a mere object-based representation provides only a tiny fraction of information needed for successful behavior in the world. This point is particularly pertinent in lower species such as rodents for whom navigation is a more immediate task than object identification (Cox, 2014). In fact, much of our visual input is not composed of well-defined objects and thus trying to parse them into objects makes little sense. A richer description is thus needed if we were to capture the essence of information about the world (Gibson, 1979).

**Figure 3:**
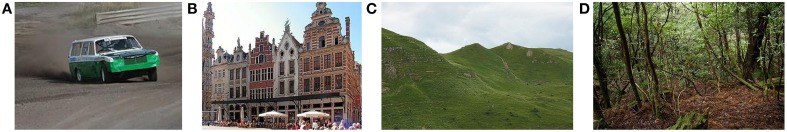
**The hierarchy of objecthood**. Objects are not the most important piece of information in every image. While **(A)** has a well-defined object, it is already less clear in **(B)** what should count as one: The row of houses? Or each house separately? Or each of the windows? In **(C)**, there are three mountains but where each of them begins and ends is neither clear nor very important, and in **(D)** layout rather than object identity dominates perception, although one can see trees, trunks, etc. (Image credits from left to right: bengt-re, 2009, Snowdog, 2005, Reza, 2009, Σ64, 2012. All images are available under the Creative Commons Attribution License or are in the public domain.).

To stress the point of the inadequacy of object-based representations, let us consider a series of images in Figure 3. In some cases, like Figure 3A, where the object (“a car”) is clearly separate (self-contained) from the rest (the road), object identification and localization provides the most important information about the scene (“there is a car”). But consider a row of buildings, for example (Figure 3B). While one still clearly describes each house as a distinct object, they are impossible to detach from other items (other houses and the ground). A more extreme example is depicted in Figure 3C, where even though a mountain is sticking out from the ground surface, it is no longer very clear where the mountain ends and the ground begins. Is the visual system really concerned about finding objects in such images then? In fact, as we go further away from close-up views into panoramic scenes, identifying objects does not appear to be the default mode any longer. In Figure 3D, we know that the image is composed of individual trees, grass and other stuff but we no longer can count them. Rather, a percept of various textures and layouts appears to dominate. Thus, talking about individual objects is largely irrelevant in these scenarios and instead describing texture properties and characteristics that allow navigation through the terrain, or a global level semantic labeling of “a forest” or “a lawn” often seems to be the more immediate task for vision (Oliva and Torralba, 2001; Torralba and Oliva, 2003).

Therefore, we point out that surfaces that mid-level areas construct are not only meant to represent the outline of objects in images but also (or primarily) to summarize the properties of textures and surfaces in the environment.

### Representations prior to identification

**Figure 4:**
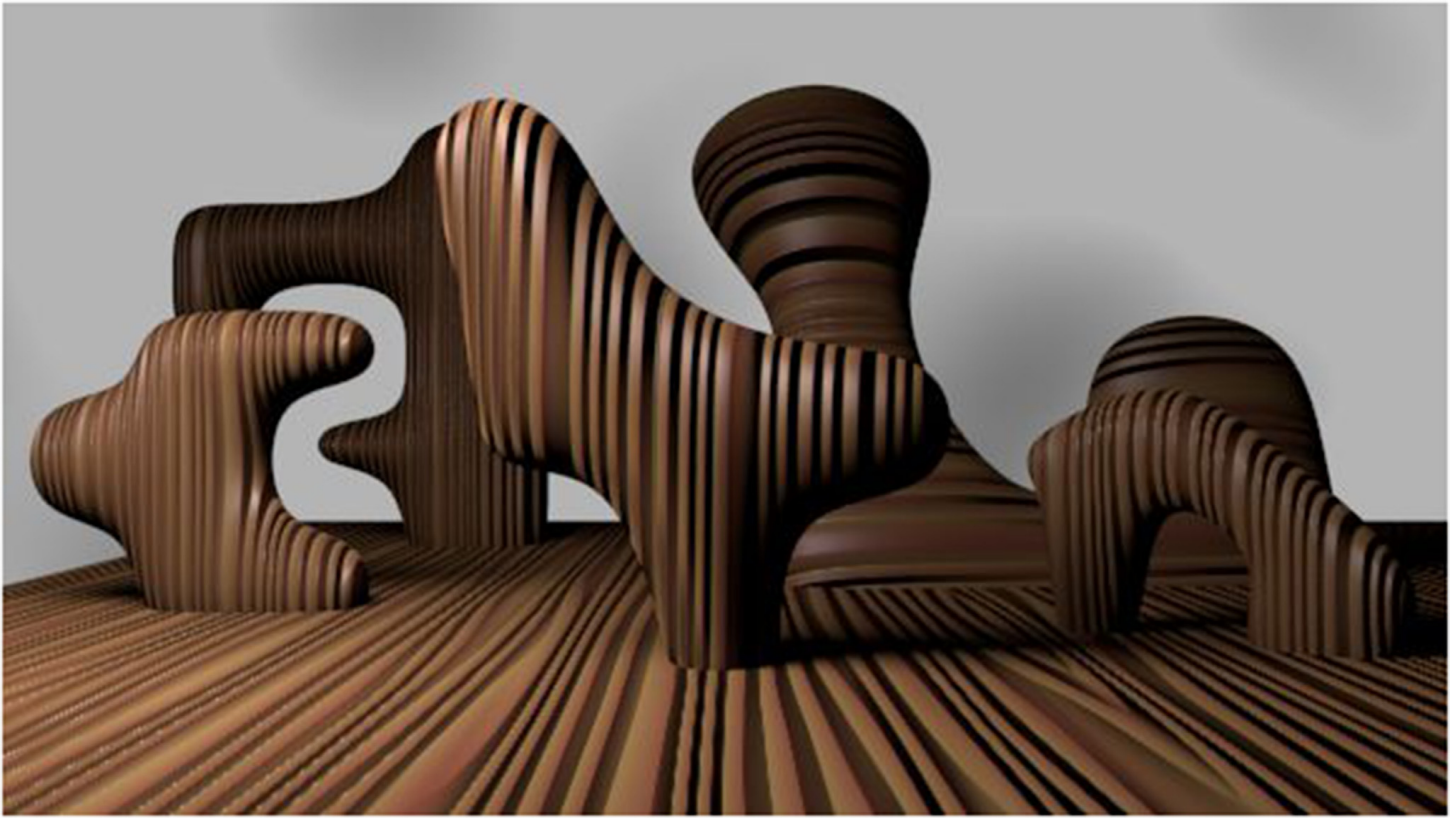
**Recognition is not crucial for scene or object understanding**. In this artificially generated scene we see five novel objects, we can describe their three-dimensional shape despite partial occlusions, and navigate around them without having to know the identity of those objects.

Finally, we point out that intermediate representations do not have to rely on being able to identify the contents, consistent with the idea that they are computed early on. We do not need to know what we are looking at to be able to describe its three-dimensional shape, texture, and spatial relations to other items in an image. For example, notice that in Figure 2 surface interpolation occurs despite us never having seen these particular shapes before and having no categorical label for them, indicating that this phenomenon could be performed by mid-level computations prior to categorization. This observation also holds for a more realistic image depicted in Figure 4, where we can easily agree that five objects situated in different depth planes are depicted. We can describe their shape and imagine acting upon them despite partial occlusions present in the image. This is clearly a more advanced representation of the image contents than a mere V1 filter output, yet not so advanced as to require any categorization, recognition or identification (naming) of the objects in it.

The idea of intermediate representations being established without recognition of contents is well-known in psychology (Witkin and Tenenbaum, 1983; Nakayama et al., 1995). To provide an illustrative example, the famous visual agnosia patient DF cannot report the identity or even orientation of most objects, yet her ability to act on these objects remains intact, a finding that has led Goodale and Milner (1992) to propose the vision-for-action and vision-for-perception division in the visual information processing in the brain. It thus appears that our visual system is adept in processing inputs even lacking knowledge about what they are, pointing to the idea that scene segmentation into objects might be more basic or more immediately performed than recognition. We do not claim that recognition is irrelevant for segmentation, as it has been shown that recognition can bias figure-ground assignment (Peterson, 1994), but our point is that it can largely be done successfully without any knowledge about the identity of objects.

### Conclusion

Taken together, we claim that the goal of mid-level areas is the construction of surface-based representations that segment the input images into objects, background surfaces, and so on, together with their textural properties, because such format of representations is sufficiently rich for the variety of high-level tasks, including three-dimensional reconstruction of the scene, navigation in it, interaction with objects or restricting attention to them. The idea of the primacy of the surface-based representation is also supported by empirical studies showing that some form of figure-ground organization would be established already shortly after feedforward inputs reach higher visual areas and is consistent with the observation that segmentation does not require knowledge of the identity of the objects involved. Importantly, given the computational complexity, this organization is probably not computed globally but rather is restricted to parts of visual inputs that fall at fixation or where an observer is attending.

It is also important to understand that the segmentation we describe here is not the same as what is commonly meant by this term. Many algorithms of segmentation only divide the image into a mosaic of non-overlapping regions without any information about the depth, that is, which region is in front of another one (see also Section “Current Approaches”). However, whenever something is occluded, that is a cue for depth ordering. Therefore, we consider a process that not only divides the image into separate regions but also infers figure-ground relations between these regions. Since this process often involves the inference of occluded parts, we refer to such interpolated regions as a surfaces.

Finally, such depth ordering is necessarily an oversimplification. For example, observe in Figure 2C that we do not perceive the whole of the black shape in front of the gray one. In fact, at least for some observers, part of the black shape (shown in red in Figure 2C) appears to be behind the gray shape, suggesting a three-dimensional form of the two shapes (Tse, 1999). This example demonstrates that the resulting representations cannot be captured by splitting an image into several depth planes, and thus require more flexibility. Such representation presumably would be followed by a full rectification of a three-dimensional volume at the later stages of visual information processing.

## Intermediate computations

We proposed that intermediate processing stages produce surface-based representations from two-dimensional static images. What computations could produce such representations?

### Current approaches

In computer vision, many early image segmentation approaches considered segmentation as a global optimization problem of finding the best boundaries, grouped regions, or both. For example, Mumford and Shah (1989) proposed a functional that estimates the difference between the original image and its segmentation with constraints for smoothness and discontinuity at region boundaries (see also Lee et al., 1992). Finding the best segmentation amounts to finding the global minimum of this functional. Similarly, in a boundary-based contour extraction model, Elder and Zucker (1996) considered finding the shortest-path cycles in the graph containing boundary elements.

However, solving for a global optimum deemed to be a complicated task, often leading to unsatisfactory results. In 2000, Shi and Malik proposed a reconceptualization of the image segmentation problem as a graph cut problem. When features in an image are represented in a graph, finding the best segmentation amounts to finding groups of features in this graph that are maximally similar within a group and maximally dissimilar from other groups. Shi and Malik (2000) showed that their normalized cuts algorithm could provide a good optimization of this criterion and, based on this approach, they later developed one of the best-known image segmentation models (Arbeláez et al., 2011; see also Felzenszwalb and Huttenlocher, 2004 and Sharon et al., 2006, for much faster implementations of this idea).

Partitioning a graph in a fixed way, however, cannot capture the inherently hierarchical structure of images (a part can be part of another part; see the windows of houses in Figure 3B), nor can it adapt to the task demands. Therefore, in recent years much effort in image segmentation research has been devoted to the development of methods for the probabilistic generation of region proposals (Arbeláez et al., 2014) that could then be refined using a higher-level task such as categorization (Leibe et al., 2008; Girshick et al., 2014; Hariharan et al., 2014) or would be flexibly reconfigured based on Gestalt principles (Ion et al., 2013).

How could such partitioning of an image graph into high-similarity clusters be implemented in a biologically-plausible architecture? Based on behavioral and neural evidence, Nothdurft (1994) hypothesized that image segmentation involves (i) suppression of responses in homogenous feature fields, and (ii) local pooling of features for boundary detection. Unlike the global optimization approaches considered above, this idea is based on completely local computations that are attractive due to their low complexity and biological plausibility. The implementation of this idea can be found in models by Grossberg (1994) and Thielscher and Neumann (2003), where texture segmentation is performed by enhancing edges that group together by the good continuation cue (using the “bipole cell” idea), and suppressing other locations in the image. Repeated over several iterations, this computation leads to the formation of the outline of the shape. This idea accounts well for Nothdurft's (1994 observations, and also provides an integrated framework of using both texture and boundary information to perform segmentation. Moreover, Thielscher and Neumann (2005) also demonstrated that this approach produces differences in convex and concave boundary appearance, in line with Nothdurft's (1994 observations.

Segmentation into distinct regions is only the first step though. As discussed in the previous section, this is not sufficient because an explicit surface construction and figure-ground relation computation need to occur as well. Some approaches (Roelfsema et al., 2002) attempted to explain figure-ground segmentation simply as an effect of increasing receptive field sizes (thus, decreasing spatial resolution) in higher visual areas. The model operates by initially detecting boundaries in the inputs and then pooling them together in higher visual areas as a result of increasing receptive-field sizes. Eventually, the whole shape is represented by a unit with a sufficiently large receptive field. Then, the figure-ground assignment can be propagated down via feedback to the early visual areas, as observed in the experiments by Lamme (1995).

However, it is unlikely that such scheme would work in more complex displays with more overlapping shapes and more variation in texture. Moreover, smaller shapes always produce higher responses in higher-level areas because their boundaries are closer together. Since these responses represent the figure-ground signal, smaller shapes are always bound to be on top of larger shapes that produce a weaker figure-ground signal. One possibility to resolve some of these issues is to use corners as indicators of the figural side. Since figures tend to be convex, the inside of a corner reliably indicates the boundary of a figure. Based on this observation, Jehee et al. (2007) proposed an extended version of the model by Roelfsema et al. (2002) that could produce more reliable border assignments.

**Figure 5:**
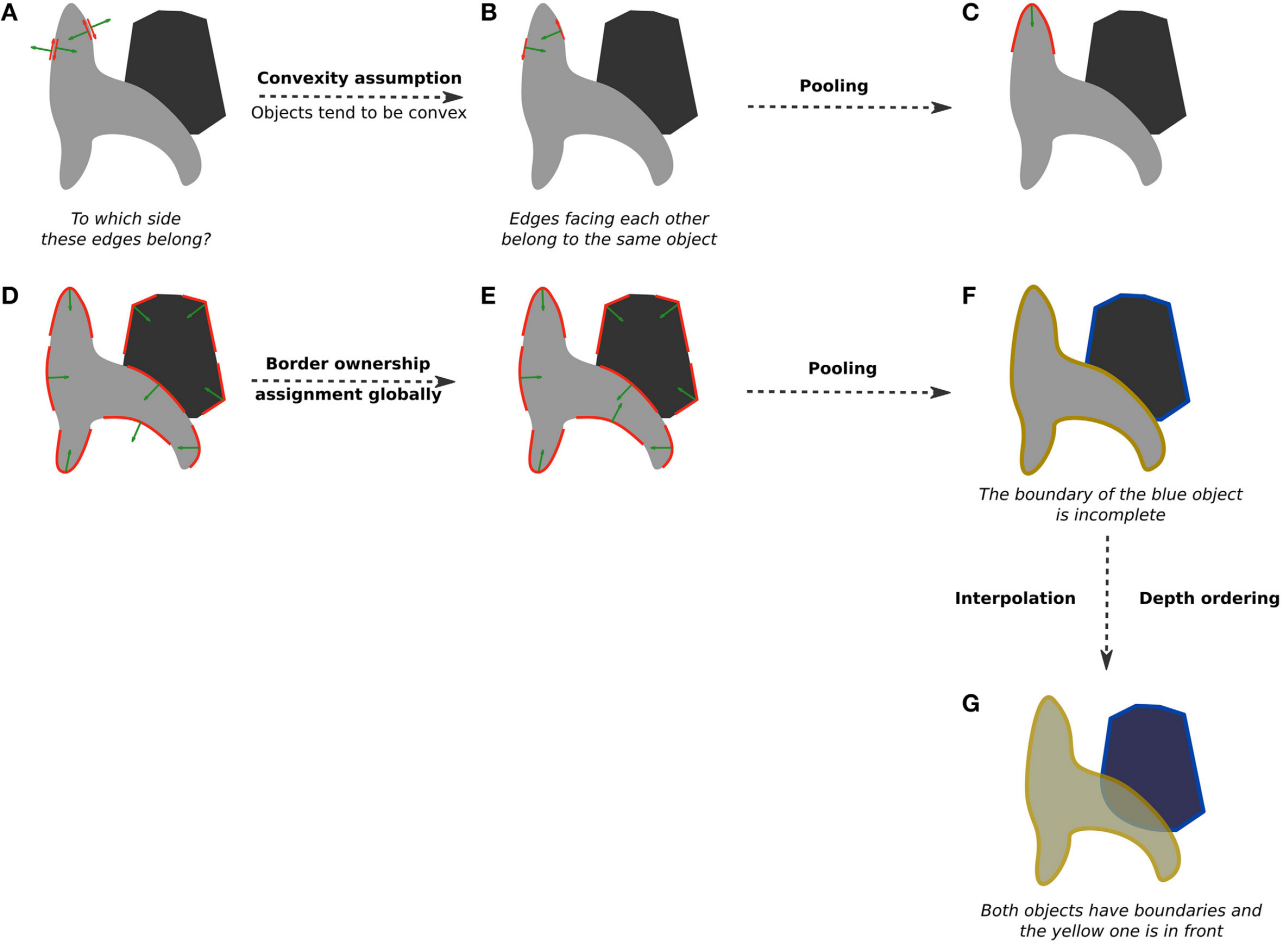
**An illustration of border-ownership assignment. (A)** Initially, each edge in the image (red arrows) can belong to one of two sides (green arrows): either the gray surface or the white surface. The goal of border ownership computation is to figure out which side is the figural side. In this case, the edges should belong to the gray surface. **(B)** Using the convexity assumption (objects tend to be convex), we can easily determine border ownership in the local neighborhood. Edges that “agree” (green arrows are “looking” at each other) are preferred. **(C)** Pooling these edges together results in a curved segment with the correct border-ownership assignment. **(D)** After this computation is carried out in the local neighborhood, border ownership is largely but not fully correct. We can improve it by using the same convexity assumption over larger areas (e.g., over the entire image). **(E)** Global border-ownership computation results in a correct assignment of all segments. **(F)** With pooling, two separate surfaces emerge. Note that the blue one is missing a boundary at the intersection with the yellow object. This implies that the blue object is partially occluded by the yellow one. **(G)** Using this information, a correct local depth ordering is established and the missing piece of the blue object is interpolated.

The idea of using convexity can be applied more generally across the entire shape outline and not only at its corners. To illustrate how that could work, consider the two shapes in Figure 5A. The two edges shown in red can either be the boundary of the gray surface or the boundary of the white one, as indicated by the green arrows pointing to both directions. Of course, in this case it is clear that these edges must belong to the gray surface because the white one is just the background. But how would a model know? If we assume that objects tend to be convex, edges that are in agreement (the green arrows that are pointing toward each other) might belong to the same surface (Figure 5B). This simple computation in the local neighborhood followed by pooling into curved segments (Figures 5C,D) results in a largely correct border ownership. If it is further computed globally over a few iterations, local inconsistencies (e.g., a concavity of the lighter gray object) can be resolved (Figure 5E; see Figure 5B in Craft et al., 2007, for a working example), resulting in the proper assignment of edges to one of the two objects (Figure 5F), which is the desired initial image division into surfaces.

Importantly, because of border-ownership, we also learn which parts of objects are occluded. If a certain surface is partially bounded by a boundary that it does not own, it is a sign of an occlusion. For example, in Figure 5F, the yellow object is partially occluding the blue one, and border-ownership assignment indicates that edges along the yellow object belong to it. That leaves the blue object lacking a closed contour, meaning that part of it is occluded. An interpolation of surface results in a more perceptually compelling segmentation into whole shapes (van Lier et al., 1994), and consequently provides an ordering of surfaces in depth (Figure 5G).

The existence of such border-ownership cells has been reported in the visual area V2 (Zhou et al., 2000; see Zucker, 2014, for a good overview) and a number of models based on this idea have been proposed since (Zhaoping, 2005; Craft et al., 2007; Layton et al., 2012). Kogo et al. (2010) extended this framework by also using L- and T-junctions to determine not only figure-ground assignment for luminance-defined figures but also to produce the correct output in the case of illusory contours (Kanizsa's figures). Importantly, unlike earlier proposals (e.g., Grossberg, 1994), their approach is capable of yielding the correct representations of comparable yet non-illusory displays without ad hoc deletion of interpolated contours (see Figure 1B in Kogo et al., 2010).

Similarly, extending their work on bipole cells, Thielscher and Neumann (2008) showed that T-junctions could be used to infer figure-ground relations for multiple figures (not just figure and ground) in their architecture, and more recently, Tschechne and Neumann (2014) extended their earlier work to a full model of figure-ground segmentation. Initially, bipole cells, curvature and corner detectors are used to produce the consistent outline of a shape. Then, contextual cues are used to compute border-ownership.

Taken together, current biologically-inspired approaches to image segmentation largely concentrate on discovering boundaries in an input image and resolving figure-ground assignment by computing border-ownership of the boundaries in an image. However, unlike purely computer vision algorithms, these approaches are typically not tested with realistic inputs, thus their applicability and robustness on the wide variety of natural images remains unclear. Moreover, some models are better at segmentation but do not perform feature interpolation and figure-ground relation computations, and vice versa, while others focus on using second-order features but are not robust for segmentation using multiple cues, and so on. In other words, each of them only implements several aspects of processes in mid-level vision but the proposed mechanisms are not mutually compatible to build a unified architecture. Could there be several basic mechanisms that could account for the majority of the available data?

### Our approach

In a nutshell, we are interested in understanding *conceptually* what computations could suffice to account for the following biologically-plausible image processing strategy:

1. Region property and boundary extraction.
2. Clustering of boundary and region features into separate surfaces (segmentation).
3. Surface interpolation and depth ordering (figure-ground organization).
4. Representation refinement via recurrent loops.

Moreover, we want these computations to be sufficiently robust such that they would apply across various features in the images and could therefore be used in the typical computer vision setups such as deep networks.

**Figure 6:**
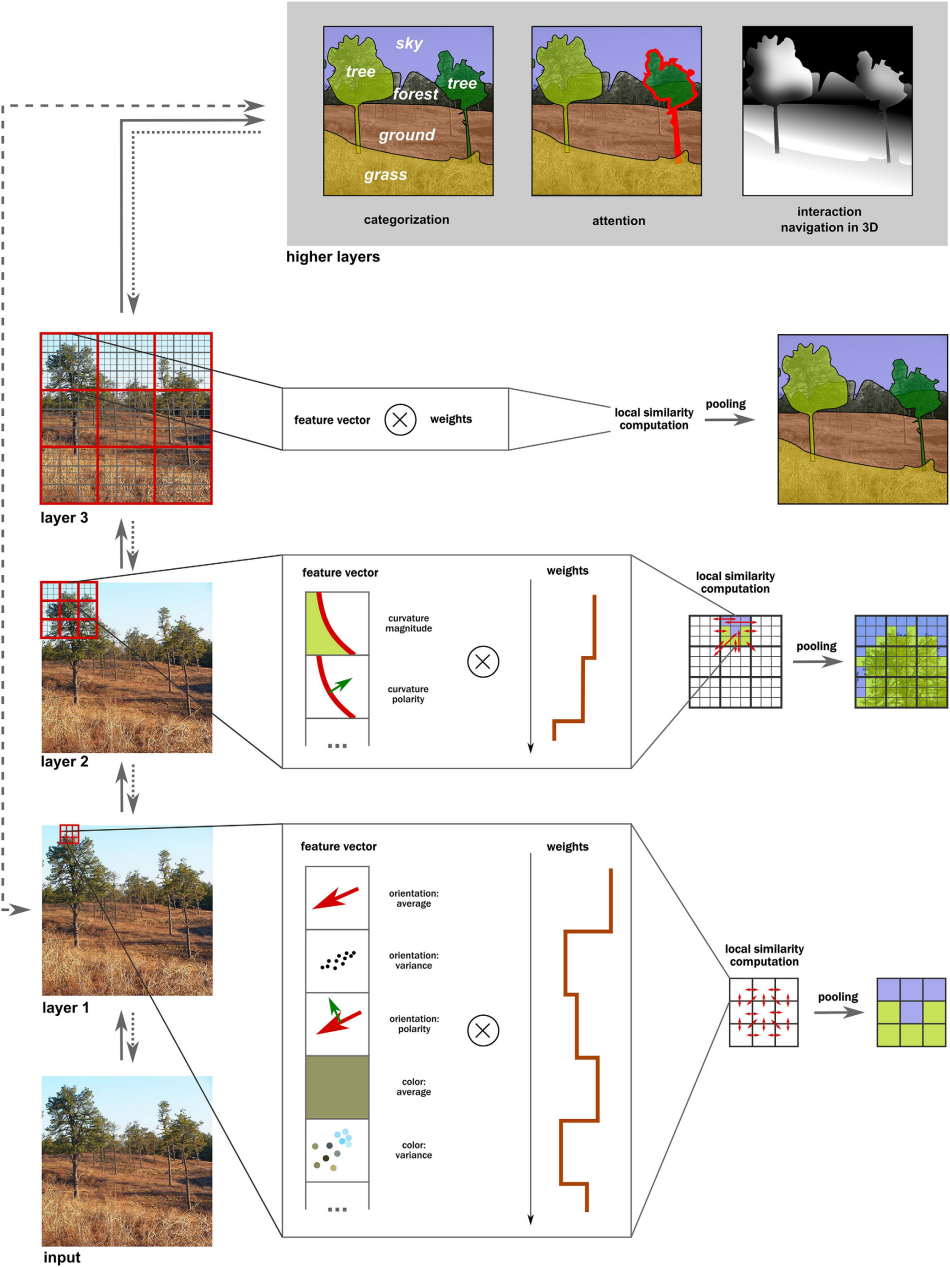
**Computation of intermediate representations in the visual hierarchy**. In each layer, various features are extracted first at each location, forming a feature vector. Next, correlations are computed in the local neighborhood between each pair of a weighted feature pair, leading to similarity statistics (red arrows). (The optimal weights need to be learned by training the model.) Finally, these patches are pooled together into clusters that contain similar statistics. These new clusters are used in the next layers for the same similarity and pooling over increasingly larger neighborhoods. Note how the resulting intermediate representations are interpolated behind occlusions and are ordered in depth (e.g., the tree is in front of the forest). These representations can now be used for higher-level tasks such as categorization, attention to specific objects or interaction with them, or for navigation. They are also rather coarse initially (e.g., trees on the right are incorrectly lumped together), and can further be refined iteratively via feedback loops (if attention is directed to that region). Moreover, notice that not all steps must necessarily be carried out as certain shortcut routes (e.g., the gist computation) using simpler statistics can occur.

To implement steps 1 and 2, we propose two basic mechanisms for intermediate computations, generalizing the vast majority of approaches discussed in Section “Current Approaches” (Figure 6):

- *similarity statistics* that compute correlations between local patches of the input, and
- *pooling* that combines together highly similar (well-correlated) patches.

These two computations are implemented hierarchically, processing over increasingly larger patches of the input image and resulting in a coarse mid-level representation of surfaces and their properties upon the first roughly feedforward processing wave. As a result of feature inference at multiple layers, the constructed surfaces are partially overlapping, providing information for depth ordering at the highest stages of this architecture (step 3). The resulting representations will be very coarse and probably inconsistent, so an iterative refinement of these representations by reapplying similarity and pooling operations over smaller parts of an input image is important as well (step 4; see also Wagemans et al., 2012b). We briefly discuss the role of feedback in Section “The Dynamic Nature of Intermediate Representations.”

### Similarity estimation and pooling

Let us start by considering the output of a typical low-level computation such as edge detection, as illustrated in Figure 5A. The red arrows in this figure show the locations and orientations of salient edges in the image. While this is a useful description of potential boundary positions in the image, this information does not suffice to understand the organization of the image contents. In particular, it does not indicate which edges are likely to define the same surface, as shown in Figure 5B. At this stage the system only knows about separate salient edge positions, and further processing is needed to group both boundary and textural elements into coherent surfaces.

Finding which edges might group together can be achieved with a simple *similarity* measure, such as a correlation between two locations in an image. If the similarity is high, the two edges might belong to the same smooth contour (since edges at nearby locations of a smooth curve have similar orientation) or the same surface composed of similarly oriented elements (e.g., the wood texture in Figure 4). In contrast, a low similarity indicates a potential discontinuity in an image, or a second-order edge, just like the one between the ground and the object in Figure 1C.

Of course, similarity computation need not be restricted to oriented edges only and can be applied across other properties (e.g., spatial frequency, phase bands, color) and even across summary statistics within a local patch (e.g., mean and variance of orientation). Notice that by incorporating multiple cues, this single computation of similarity among the adjacent locations provides a natural approach to dealing with both boundary and textural cues in images. In particular, wherever there is sufficient dissimilarity, textural properties are actively used to generate boundary elements that are further used to construct full surface boundaries.

Freeman et al. (2013) provided evidence that such similarity measures are indeed computed early in the visual system. They constructed synthetic textures with specific higher-order statistical dependencies, such as marginal statistics, local cross-position, orientation, scale and adjacent-phase correlations, and demonstrated that such neurons in primate V2 (but not V1) were particularly sensitive to these built-in statistics, suggesting that V2 computes similarity between features. When used in textures, such summary statistics apparently are sufficient for the synthetic generation of similar-looking textures (Portilla and Simoncelli, 2000). When used on natural images, these statistics appear compatible with percept in peripheral vision (Freeman and Simoncelli, 2011; Freeman et al., 2013) and can also account for certain effects in crowding (Balas et al., 2009) and visual search (Rosenholtz et al., 2012).

Similarity statistics alone are not sufficient, however. While they are clearly useful in providing rich descriptions of the inputs, the number of parameters in the system increases dramatically since these statistics are computed pairwise between many small patches. Maintaining all these parameters does not appear to match our phenomenology where integrated shapes or regions dominate over local fragmented interpretations. Moreover, natural scenes contain substantial redundancy and the visual system appears to take advantage of it via efficient coding strategies (Attneave, 1954; Barlow, 1961; Simoncelli and Olshausen, 2001; Olshausen and Field, 2004; DiCarlo and Cox, 2007). For instance, Vinje and Gallant (2000) demonstrated that V1 neurons use a sparse encoding scheme that matches the sparse structure of natural scenes. Other researchers have demonstrated that sparsity constraint leads to the development of simple and complex cells in computational models (Olshausen and Field, 1996; Hyvärinen and Hoyer, 2000, 2001).

It thus appears that a higher-order statistic, one that would summarize similarity statistics, is necessary. We call this computation *pooling* to reflect the idea that separate units are now pooled together according to the strength of the previously computed pairwise correlations. Computationally, such pooling operation is very simple, for example, a single-link agglomerative clustering of patches that correlate above a certain threshold (Coates et al., 2012) or mean-shift (Paris and Durand, 2007; Rosenholtz et al., 2009). The threshold can be flexible (i.e., a free parameter in the model) reflecting individual differences between participants.

While either similarity or pooling have been utilized in various formats separately by many models, exploring the power of their combination is rare. Geisler and Super (2000) showed that a similar similarity and pooling scheme could account for a number of typical perceptual grouping displays. One successful demonstration of this combination on real images was reported by Yu et al. (2014) who found that a super-pixel segmentation followed by mean-shift clustering accounted surprisingly well for visual clutter perception. In a notable example that such scheme can be both powerful and efficient even for practical applications (due to parallelization), Coates et al. (2012), using *K*-means and agglomerative clustering, achieved robust unsupervised learning of face features using tens of millions of natural images.

### Hierarchical similarity estimation and pooling

While it would be possible to perform similarity and pooling globally across the whole image, such strategy would be very inefficient and probably not very accurate. Instead, we propose that these computations are performed hierarchically, such that first similarity and pooling are done locally, then over somewhat larger neighborhood using the newly inferred features, and finally globally using few but rather complex features that result from these computations at earlier stages.

**Figure 7:**
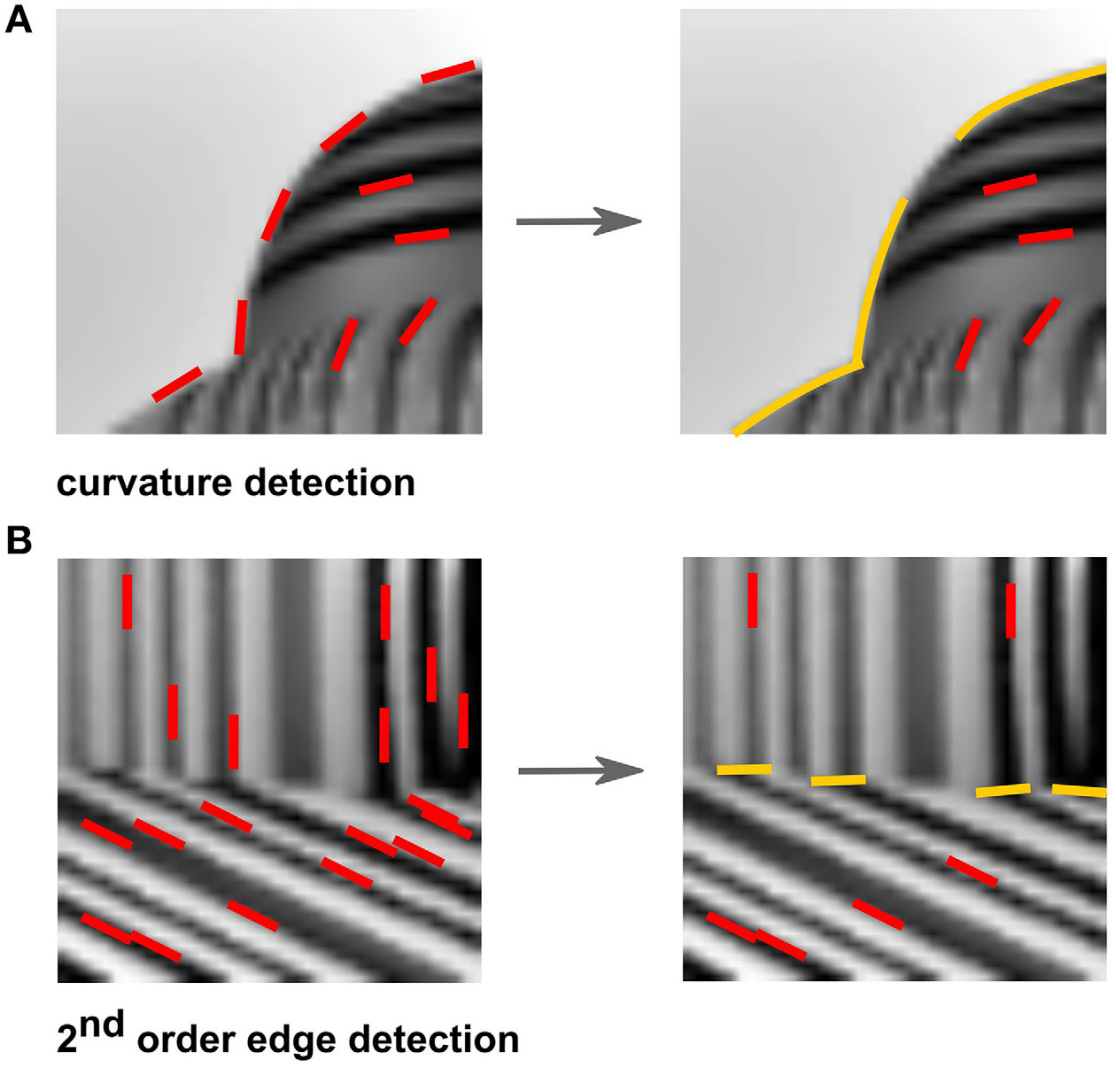
**Examples of feature inference. (A)** Pooling similarly-oriented features (red) result in the inference of curved fragments (yellow). **(B)** In contrast, a dissimilarity between oriented features (red) result in the inference of second-order edges (yellow) between textures.

The initial computation of a similarity and pooling would yield longer straight or curved segments (Figure 7A, right). A low correlation, on the other hand, would indicate the presence of second-order edges that are formed between adjacent surfaces with differently oriented elements. For example, in Figure 7B, left, there is no clear edge separating the object from the ground since their overall luminance is quite similar, and thus segmentation could not be done with a simple V1-like edge detection model. The desired segmentation becomes trivial when the difference in orientation content is observed. The dominant orientation of the object is different from that of the ground and can therefore be used to determine a boundary between the two textures, which is indicated by the low similarity measure (Figure 7B, right).

Of course, detecting second-order edges in this fashion can also yield spurious results. Boundary element orientation can change significantly at inflection points (i.e., junctions) leading to low similarity measures, and yet these do not imply the presence of a second-order edge. One solution to the problem could be to use only sharp edges for defining boundaries, and otherwise assume that edges define textures (the insides of a surface). Consistent with this idea, Vilankar et al. (2014) reported that edges defining an occlusion tend to have steeper changes in contrast than non-occlusion edges (reflectance difference, surface change, cast shadows) and that a maximum likelihood classifier could predict the type of edge with 83% accuracy in their database. Another possibility is that junctions are not detected during the initial processing and only computed later when the global estimate of the shape is already available from the higher-level areas. Consistent with this idea, McDermott (2004) reported that participants were unable to report T-junctions using local natural image information (small patches of image) only (but see Hansen and Neumann, 2004; Weidenbacher and Neumann, 2009).

However, in general, the visual system is not so much interested in the features as such but in the surfaces they define. Other cues than boundaries can therefore be important in the local computations of which features should be combined into a single surface. As discussed above, convexity is an important cue for border-ownership assignment. Measuring consistency in edge polarity (where the brighter side is) can also provide information if they are likely to belong together (Kogo and Froyen, 2014). In fact, Geisler and Perry (2009) observed that edges with an inconsistent polarity are less likely to belong to the same contour. Recently, it has been reported that even low-level cues, such as the sharpness of an edge or local anisotropies in spectral power can be informative about figure-ground organization (Ramenahalli et al., 2014; Vilankar et al., 2014).

So, at each location where a boundary element has been found or inferred, we can list all these cues as a long vector and then compute the similarity between these vectors in the local neighborhood. Sufficiently similar locations are then pooled together, resulting in new, more complex features at a higher layer of this hierarchy. Now again, the similarity of these new features over larger scales can be computed, and similar features pooled together into even more complex features, such as parts of boundary (Brincat and Connor, 2006) or surface patches (Yamane et al., 2008) with a complex geometry. Finally, these features are pooled again over the entire image, producing the initial segmentation of an image into proto-surfaces.

### Neural representation of pooled units

By definition, a pooling operation combines outputs of several units and treats them as belonging to the same group (same contour, shape, or surface). Several alternatives have been proposed how such groups could be represented in the visual system. Perhaps the most straightforward way to implement this representation is by having dedicated grouping cells. Such idea has been used in a computational model of border-ownership assignment by Craft et al. (2007). They implemented neurons with donut-shaped receptive fields that can pool together units lying on that donut. However, such grouping cells have yet to be found in the visual system. It is possible however that cells with large curved receptive fields that exist in V4 might suffice to perform the border-ownership computation (as the authors themselves suggest on p. 4320 of their paper).

Another simple strategy is an increase of the mean neural response of units belonging to the same group (Roelfsema et al., 2004). However, this strategy also implies that only a single group can be maintained at a time. If another group needs to be processed, such as when shifting attention from one object to another, the integration computation would have to be performed again. While it may appear somewhat limiting, it should also be noted that in many tasks, such as multiple object tracking, observers show a rather poor ability to maintain representations of multiple groups at the same time.

A very different idea has been proposed by von der Malsburg (1981). He hypothesized that representations are held together by synchrony in neuronal firing. Such idea, if true, would in theory allow for multiple stable representations to co-exist in the visual system. While such synchrony has been observed in the visual cortex (Singer and Gray, 1995), its functional role is heavily debated, questioning whether it indeed plays a causal role in representing groups (Roskies, 1999; Roelfsema et al., 2004).

Finally, a similar idea has been put forward by Wehr and Laurent (1996). They provided evidence that locust's olfactory neurons fire in a certain unique temporal patterns to various combinations of scents. For example, while an overall response to an apple and to a mint and an apple scents might appear comparable, at a finer temporal scale differences emerge in the number and timing of these higher frequency peaks (three peaks for the apple scent but only two for mint and apple). In other words, each stimulus receives a unique code of neural firing which can serve as a tag for belonging to a certain group. Importantly, just like binary code in computers, this code can accommodate a large number of stimuli without running into the combinatorial explosion.

### The dynamic nature of intermediate representations

The visual processing need not stop with the feedforward formation of the intermediate representations. Probably the best we can expect at this first pass of processing is a very coarse representation capturing the most salient aspects of the input. For example, the initial representations may lack global consistency: it is likely that not all parts of an object will be bound into a single entity, and there can also be errors of the bounding of parts. For instance, the legs, body, and arms of a human body might be separate initially if there is not enough similarity between them. As a result, these parts may also have conflicting figure-ground assignments, such that the body is computed to be behind a chair but the legs are in front. If necessary for the task, a reconfiguration of these components could be formed iteratively until a global minimum is found, resulting in a stable percept of the configuration. For instance, the border-ownership model by Zhaoping (2005) resolves the direction of border-ownership by iteratively computing which side is more likely to be the figural side. The iterations are necessary because, for example, borders in concave parts of a shape might initially have the wrong border-ownership (toward the convex side) but over several iterations the assignment is gradually reversed since other parts of the global shape influence the decision that the concavity should be part of the whole shape. There are also cases where several interpretations are similarly plausible (e.g., the Necker cube or the vase-face figure; see Wagemans et al., 2012a), and thus iterative computations will lead to continuous switches between these interpretations.

In many cases, the refinement of representations will also be necessary. In particular, the initial representation formed in mid-level areas might only capture the gist of the input. Details will be necessarily lost due to agglomerative pooling operations. In order to extract finer details, representations in earlier layers can be reaccessed via feedback loops (indicated by backward arrows in Figure 6), as conceptualized by the Reverse Hierarchy Theory (Hochstein and Ahissar, 2002). Such feedback connections are abundant in the primate visual cortex and have been implicated to be important for various purposes (Felleman and Van Essen, 1991; Angelucci et al., 2002; Roelfsema et al., 2010; Arall et al., 2012). For example, intermediate representations could be used as saliency maps to direct attention to a particular part of an image or a particular feature (Walther and Koch, 2006; Russell et al., 2014). Then irrelevant inputs would be inhibited while the relevant ones would receive an enhanced weight (Mihalas et al., 2011; Arall et al., 2012; Wyatte et al., 2012), and the whole similarity and pooling computation would be repeated again. Such approach could be particularly important for resolving complicated parts of images that require high spatial resolution (Bullier, 2001), serial (or incremental) grouping of image features (Roelfsema, 2006), and could play a major role in learning features from input statistics (Roelfsema et al., 2010).

Iterative computations also provide the necessary flexibility for dealing with the inherently hierarchical composition of scenes. Consider, for example, Figure 3B, where all buildings could be represented by a single surface, or could be further divided into separate surfaces for each building, or even further for each window or any other detail in the image. Task demands, the mental state of an observer, and other factors can have a strong influence to the percept at any given moment. Utilizing the recurrent connections, the dynamics of the percept could be modeled in our framework by updating the pooling threshold (Sharon et al., 2006; Ion et al., 2013).

Of course, the proposed system need not be strictly hierarchical. For certain computations, it makes sense to have fast bypass routes (indicated by the dashed arrow at the top of Figure 6) whenever construction of intermediate representations is too slow or unnecessary, as could be the case for face detection where Viola and Jones' (2001) approach proves sufficient, or for a rapid scene categorization using the gist computation (Torralba and Oliva, 2003). Moreover, including such bypass routes naturally provides the visual system with the flexibility to both build detailed representations gradually and also to produce global impressions of the input statistics rapidly (Bar, 2004). The gist of the scene can provide informative priors (category, context, memory associations, and so on) that could guide processing and segmentation at intermediate layers (Peterson, 1994; Rao and Ballard, 1999; Oliva and Torralba, 2007).

Finally, we want to stress that although recurrent processing can improve surface representations and help in task performance, figure-ground segmentation does not require it. For example, Supèr and Lamme (2007) observed that removing most of feedback connections from higher visual areas to V1 reduced but did not abolish figure-ground perception. In fact, Qiu et al. (2007) reported that border-ownership signals emerge pre-attentively, and a purely feedforward model of figure-ground segmentation has been proposed by Supèr et al. (2010), consistent with a limited role of feedback in figure-ground assignment process (also see Arall et al., 2012, and Kogo and van Ee, 2014, for a discussion).

## Evaluating performance

The proposed architecture is meant to simulate the representations residing in mid-level vision. Given that this is not the final stage of the visual processing, evaluating the model's performance is not trivial. Often, models of vision are evaluated using standard object identification or scene segmentation datasets such as the ImageNet (Deng et al., 2009) or the Berkeley Segmentation Dataset (BSDS500; Arbeláez et al., 2011), where the goal for a model is to produce labels or segmentations as close as possible to the correct answers defined in that dataset. So, one simple solution for testing our architecture could be to extend it to perform one of these tasks. In this section, however, we discuss how blindly applying standard benchmarks can be misleading and highlight the need for good, carefully constructed tests and datasets that would help to detect shortcomings in the model and guide its development (Pinto et al., 2008).

**Figure 8:**
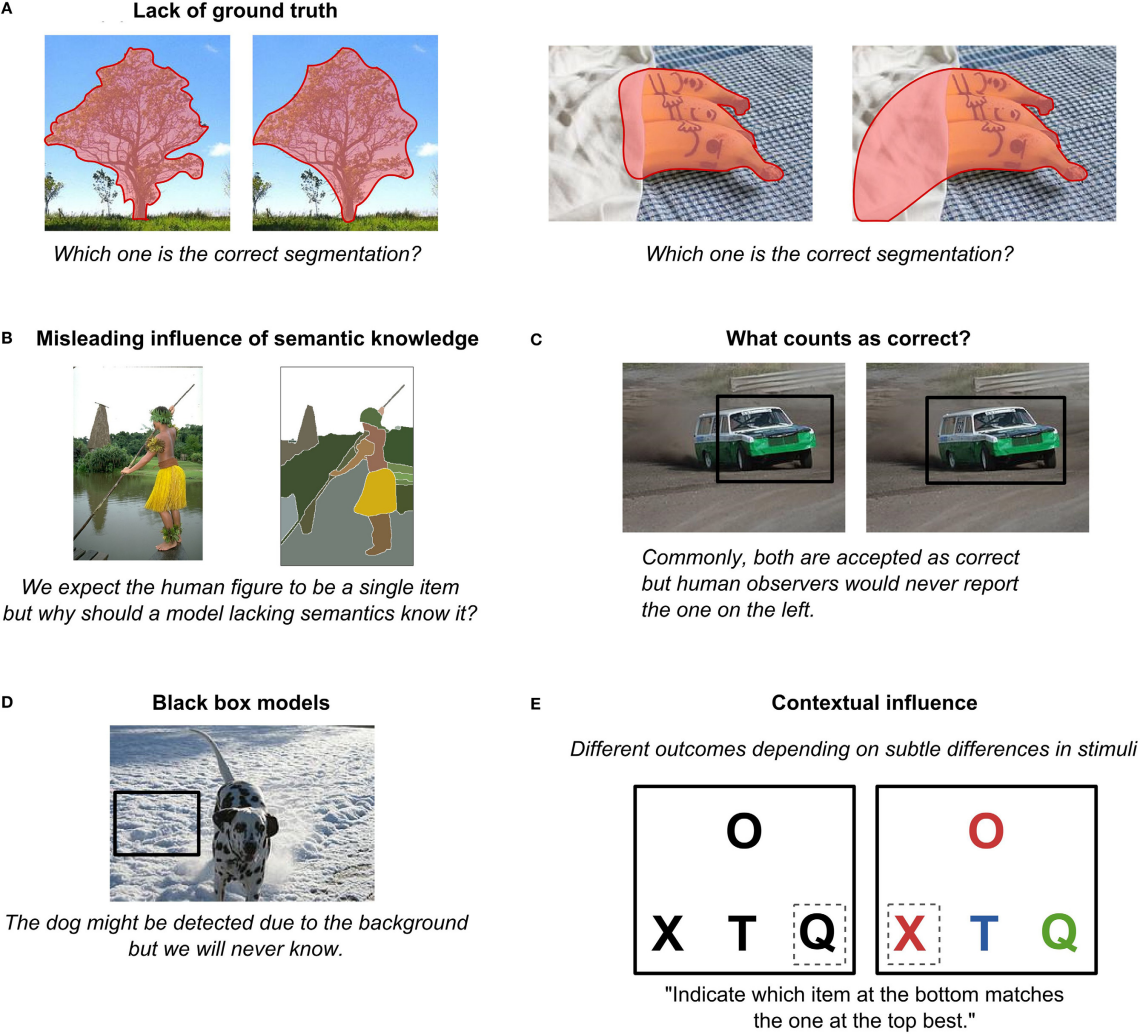
**Several potential problems with image datasets**. **(A)** Lack of ground truth (photo credits: Sheila in Moonducks, 2010, and Camera Eye Photography, 2013). **(B)** Misleading influence of semantic knowledge (image from the dataset described in Arbeláez et al., 2011). **(C)** What counts as correct? (photo credit: bengt-re, 2009). **(D)** Black box models (photo credit: Berbezier, 2008; inspired by Landecker et al., 2013). **(E)** Contextual influence. All photos are available under the Creative Commons Attribution License.

First of all, there is always the question of the “ground truth.” For example, which of the two segmentations in Figure 8A, left, is the ground truth? Both seem reasonable to a human observer and, in fact, they have been annotated by hand, making them, by definition, not objective. For example, smaller objects might be missing, subordinate categories might remain not annotated, and there may even be a disagreement among raters as to what constitutes an object and what is only a part of an object. While it is possible to step away from human raters altogether by obtaining ground-truth data using motion and depth information (Scharstein and Szeliski, 2002), only obtaining more precise measurements is not solving the major issue. In particular, the differences in ratings are largely driven not by imprecise annotation of boundaries but rather reflect individual differences in how people perceive images and what task they think they need to do. In other words, there is no ground truth to natural images because, as we have repeatedly pointed out in this paper, perception (and thus the definition of objects) is observer- and task-dependent. Another pertinent example to illustrate this point are images that contain occlusions (Figure 8A, right): What sense does it make to ask about the ground truth if it could be anything behind this occlusion, and we will never be able to tell from the incomplete data in the image? It only makes sense to ask what it looks like to a particular observer, so by forcing models to match the “ground truth,” we may in fact be pushing them to solve the wrong problem.

Similarly, raters are subject to their semantic knowledge. A human figure in a yellow skirt (Figure 8B) might be annotated as a human figure rather a body and a skirt separately. But for a model lacking extensive semantic knowledge (or statistical co-occurrences of higher-level entities), there is no reason why that yellow blob that happens to be a skirt could not be an occluder, unrelated to the human (like a flying broomstick). Regardless of whether or not the model combines the two into a single object, it does not mean that the model performed an incorrect initial segmentation. Thus, one needs to be very careful when defining what a correct segmentation is for a given model. A ground truth for one model might not be a ground truth for another.

Perhaps due to the lack of the ground truth, object localization is usually treated as accurate if at least 50% of the box containing the object overlaps with the box proposed by the model (Russakovsky et al., 2013). While finding the bounding box can often provide a good first guess of an object's location, as discussed in Section “Feature Interpolation,” it is clear that this measure is far from the explicit human knowledge of the precise boundary and location of an object (Figure 8C). As a result, a model that is performing well according to this benchmark might be doing so in a completely different way than we expect or want. For example, an interesting study by Landecker et al. (2013) attempted to track down which parts of an image end up being most important for classification in hierarchical networks. Curiously, they found that sometimes object classification decision was based on completely irrelevant information, such as a background whose statistics happened to match certain object characteristics (Figure 8D). Szegedy et al. (2013) provided another striking example where they showed that in a standard deep learning setup for every image it was possible to construct another perceptually indistinguishable image that would nevertheless be categorized incorrectly by the same network. Similarly, analyzing top-performing models in the Image Net Large Scale Visual Recognition Challenge 2012, Russakovsky et al. (2013) observed that while such models tend to provide rather accurate locations of detected objects, their performance deteriorates significantly with more objects or clutter. If object shapes were explicitly represented, clutter would play a much smaller role in localization errors. Finally, Torralba and Efros (2011) showed that models trained on one dataset often perform poorly on another dataset for the same categories of objects. What these models are learning then remains rather questionable. (However, note that there are also examples of models that are capable of generalizing across datasets; see Razavian et al., 2014.)

Finally, a model's output is extremely context dependent. For example, imagine that you are presented with a screen with one stimulus at a top and three below, as in Figure 8E, left. You are asked to indicate which item at the bottom matches best the one at the top. Most people would probably choose “Q.” But now imagine the stimuli were slightly changed (Figure 8E, right). Most people would now go for “X.” But how would a model know that? It should somehow take it into account that the colors of “O” and “X” match while “T” and “Q” have some other colors and it should also know that color is more important to the visual system than shape. In other words, it needs a lot of basic knowledge, or basic reasoning skills, that are arguably even harder to build in the system than vision itself.

To avoid some of the listed problems, we suggest using artificially generated scenes, such as the one in Figure 4. They can be rendered to contain many difficult features that are abundant in natural images, including shadows, occlusions, clutter, and realistic textures. However, unlike natural images, such scenes do have a well-defined ground truth because they are rendered from three dimensional models. Moreover, since they lack known objects, a good model should be completely capable of dividing an image into surfaces all on its own with little or no mistakes. If the model fails, it is a clear indication that intermediate representations are not being constructed properly yet.

Another possibility to evaluate model's performance is to use the extracted statistics to synthesize new images. This approach was taken by Portilla and Simoncelli (2000) who convincingly showed that their texture synthesis model was accurate by presenting an original texture and synthetically generated ones using the computed statistics. Arguably, such approach would be much trickier to implement for a synthesis of objects (Portilla and Simoncelli's procedure fails to produce coherent objects) but then the model's performance would be more directly observable and would point to issues where the algorithm needs an improvement.

## Limitations and conclusion

In this paper, we provided a synthesis of the classical works in psychology and recent advances in visual neuroscience and computer vision into a single unified framework of mid-level computations. We hypothesized that two basic mechanisms, namely, similarity estimation and pooling, implemented hierarchically and reiterated via recurrent processing, appear to be sufficient to account for the computational goals of mid-level vision and the available empirical data.

Admittedly, many details in the proposed framework remain speculative at this point. While we provided the sketch of each processing stage (including the initial feature extraction, junction and curvature computation, region growing, border-ownership assignment, and figure-ground organization), it remains to be seen to what extent these computations are robust in natural image processing. Similarly, while the framework can flexibly operate in various feature spaces, we do not propose which features in particular should be included and how different cues could be combined. Learning the weights of these cues is crucial if we want the proposed framework to apply for real images. One possibility is that the proposed computations can be implemented in the standard deep learning networks (by replacing non-linearity and normalization steps with similarity estimation, and also performing feature inference instead of a simple filtering).

Another possibility, given that, unlike deep networks, the proposed architecture does not require semantic knowledge to be trained, observing certain feature co-occurrences (see Geisler, 2008, for a review) would be a simpler way to learn and adjust these weights. Even more powerful cues would be available from dynamic or stereo-defined inputs, given their tremendous role in bootstrapping the visual system (Ostrovsky et al., 2006, 2009)

Furthermore, we restricted the scope of our discussions to the construction of the initial figure-ground organization briefly after stimulus onset. This choice has been motivated by our interest to advance the idea that image segmentation and figure-ground organization might be rapid, nearly feedforward computations. However, recurrent processing loops are undoubtedly necessary to improve the constructed surfaces and meet task demands. We considered several alternatives for such computations in Section “Evaluating Performance,” but the details of such top-down refinement remain to be worked out.

More than anything, this paper is our manifesto on the importance of intermediate computations. We are calling for a reconsideration of the role of mid-level vision and propose that implementing several basic mechanisms might provide an significant step forward in understanding the functioning of primate visual system.

### Conflict of interest statement

The authors declare that the research was conducted in the absence of any commercial or financial relationships that could be construed as a potential conflict of interest.
